# Extrauterine Leiomyoma of the Sigmoid Colon Mimicking an Adnexal Mass

**DOI:** 10.7759/cureus.89842

**Published:** 2025-08-11

**Authors:** Collin N O'Dell, Joseph S McCadams

**Affiliations:** 1 General Surgery, A.T. Still University - Kirksville College of Osteopathic Medicine, Kirksville, USA; 2 General Surgery, Mercy Hospital Southeast, Cape Girardeau, USA

**Keywords:** adnexal mass, extrauterine leiomyoma, mesentery, pelvic mass, sigmoid colon

## Abstract

Leiomyomas are benign smooth muscle tumors that typically arise in the uterus but can also occur in less common locations such as the cervix, vagina, broad ligament, or gastrointestinal tract. Extrauterine leiomyomas are rare and can be difficult to localize with imaging. We present a case of a 38-year-old woman with a left pelvic mass that was initially suspected to be ovarian in origin based on computed tomography and ultrasound images. However, diagnostic laparoscopy revealed a well-circumscribed mass originating from the sigmoid colon mesentery. Final histopathological analysis confirmed the mass to be a benign extrauterine leiomyoma. This case highlights the diagnostic challenge of differentiating pelvic masses and underscores the importance of maintaining a broad differential diagnosis when evaluating adnexal masses.

## Introduction

Leiomyomas, or fibroids, are benign smooth muscle tumors most commonly found in the uterus [1]. While uterine leiomyomas are well characterized, extrauterine leiomyomas are rare and not well defined in incidence. When these extrauterine leiomyomas occur, they can mimic other pelvic or abdominal masses, making diagnosis challenging [2]. Pelvic masses are typically evaluated using ultrasound and MRI imaging, with an initial focus on ovarian, tubal, or uterine pathology [3]. However, extrauterine leiomyomas, particularly those involving the sigmoid colon, can closely resemble adnexal masses, leading to potential misdiagnosis [3,4]. We report a rare case of a large extrauterine mesenteric sigmoid colon leiomyoma that mimicked an adnexal mass on imaging. 

## Case presentation

**Figure 1:**
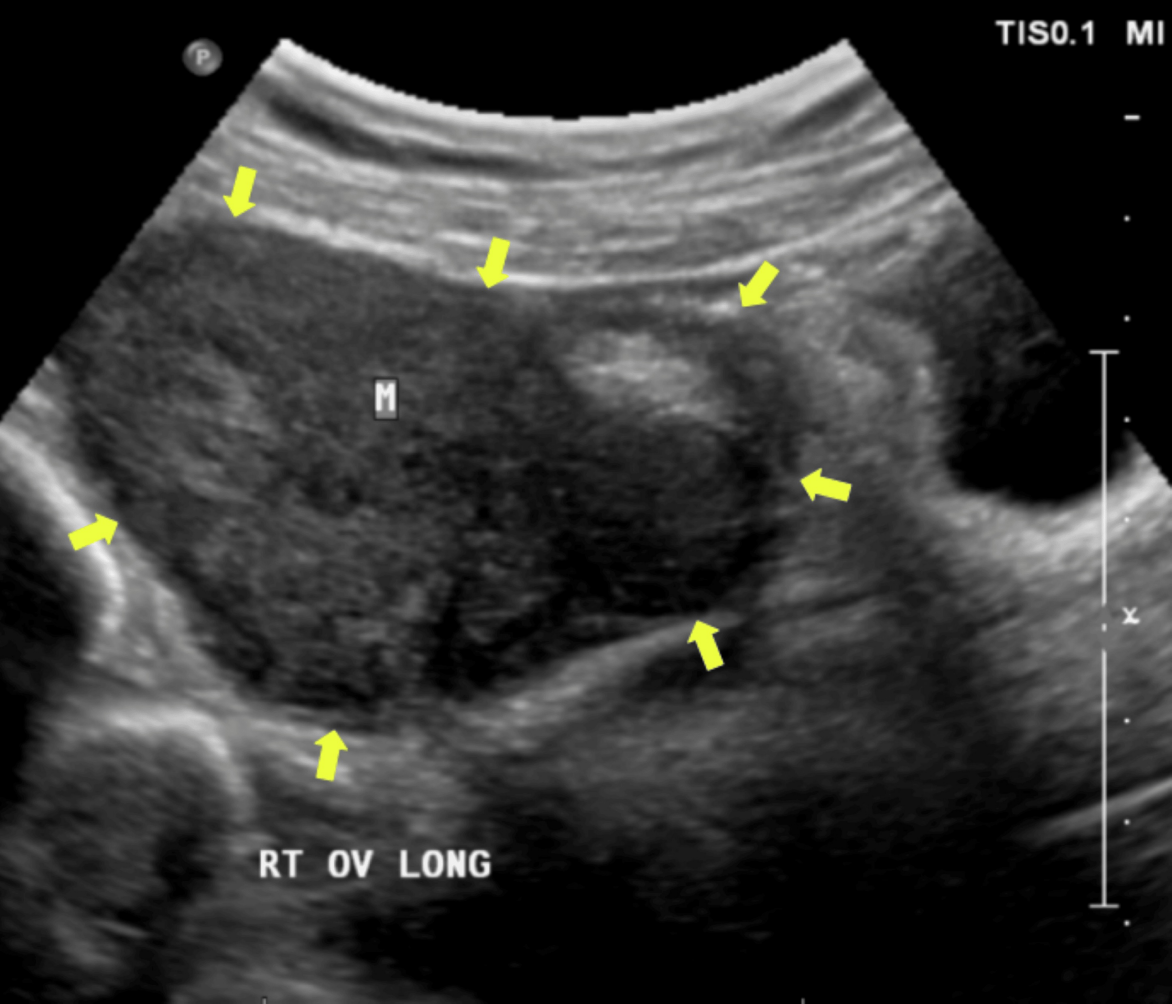
An ultrasound showing the leiomyoma in a longitudinal view. Yellow arrows showing border of leiomyoma. M - Leiomyoma

**Figure 2:**
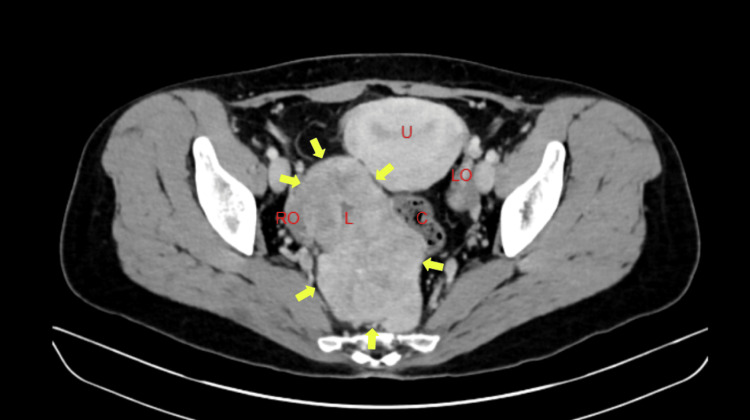
A CT Abdomen/Pelvis showing leiomyoma in transverse view. Yellow arrows showing leiomyoma border. U - Uterus, RO - Right Ovary, LO - Left Ovary, L - Leiomyoma, C - Colon.

**Figure 3:**
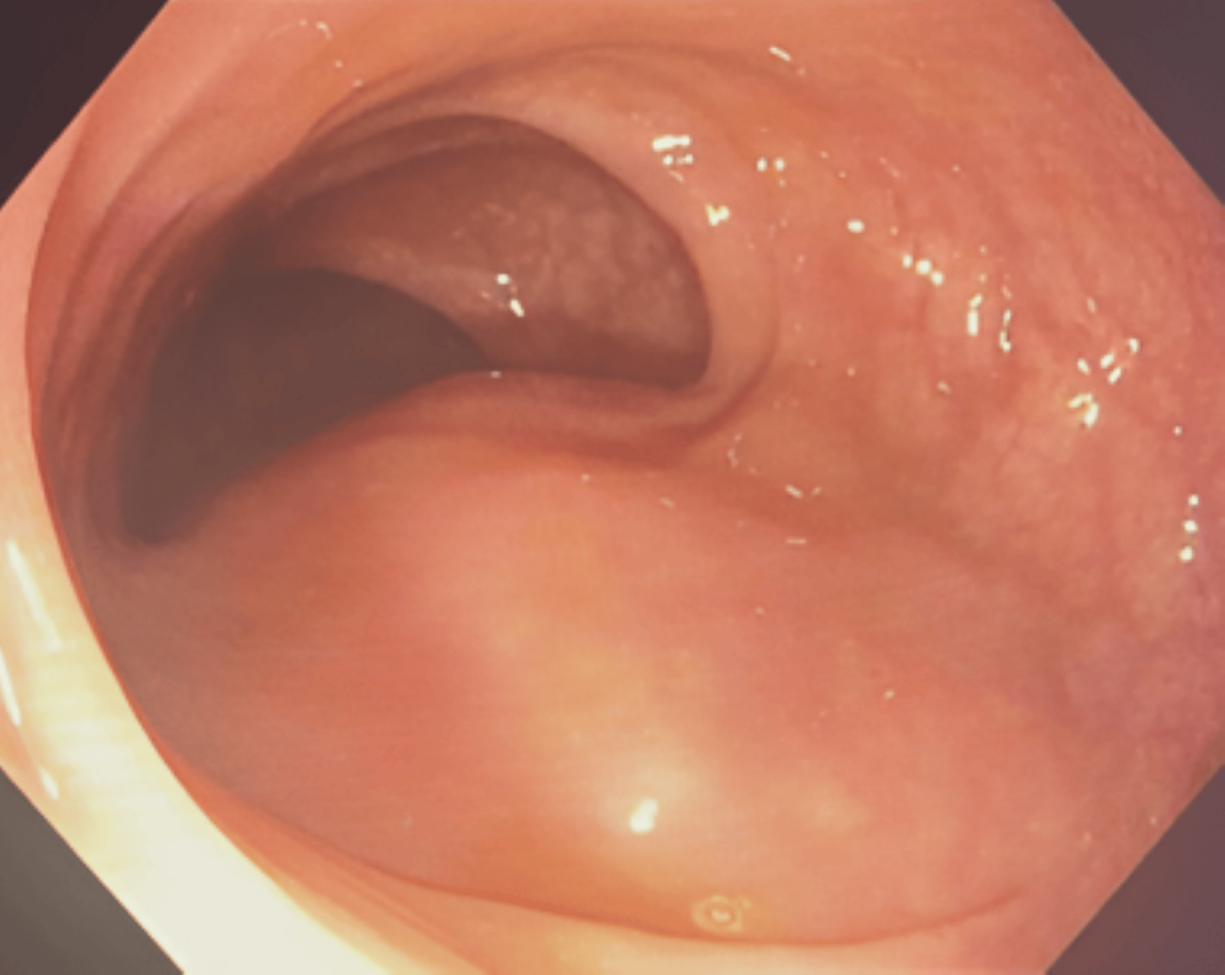
Colonoscopy showing external compression of the sigmoid colon lumen.

A 38-year-old G1P1 female presented with bilateral lower abdominal pain for two weeks. The pain was dull and constant, with no identifiable causes or relief. She also experienced decreased appetite but denied changes in bowel habits, nausea, vomiting, or urinary symptoms. Her last menstrual period was three weeks ago. Medical history included chronic diarrhea treated with Lomotil and dysmenorrhea, with a previous cesarean section. She abstained from tobacco, alcohol, and recreational drugs. Family history included maternal diabetes. Physical examination showed a well-appearing patient with normal vital signs: temperature 99.3°F, blood pressure 125/75 mmHg, heart rate 115 bpm, weight 62 kg, and oxygen saturation 98%. Abdominal examination revealed tenderness in the right lower quadrant, left lower quadrant, and suprapubic region, without guarding or rebound tenderness. Cardiopulmonary findings were normal. Laboratory studies, including complete blood count, complete metabolic panel, urinalysis, and pregnancy testing, were all normal. Ultrasound showed a small posterior fibroid and a solid right adnexal mass measuring 11.6 × 6.1 × 6.4 cm with internal vascularity (Figure 1). The left ovary was normal. A gynecologic exam confirmed a right adnexal mass in the posterior cul-de-sac, and serum CA-125 was 16.5 U/mL, within normal limits. CT imaging revealed a solid, heterogeneous right adnexal mass (11 × 6.2 cm) without uterine involvement (Figure 2). Diagnostic laparoscopy identified a smooth, solid mass arising from the lower anterior sigmoid colon mesentery, with two endometriotic lesions and multiple adhesions. Colonoscopy confirmed extrinsic compression of the colon at 15 cm from the anal verge, consistent with the mass seen during laparoscopy (Figure 3). The patient underwent laparoscopic lower anterior resection with bilateral ureteral stent placement. A 13 × 8 cm lobulated mass, involving the distal sigmoid colon and proximal rectum, was excised and sent for pathology. The initial pathology report classified the mass as a spindle cell neoplasm, leading to referral to the Mayo Clinic for further evaluation. Subsequent histopathological analysis confirmed the mass to be a benign leiomyoma. Postoperative recovery was uneventful, and the patient was discharged on postoperative day two.

## Discussion

This case highlights the challenge of distinguishing adnexal masses from tumors that originate outside the reproductive system using standard imaging. The initial ultrasound and CT scans suggested an adnexal mass, but neither clearly showed that the mass was arising from the sigmoid colon mesentery. These limitations are well recognized in the literature. Studies show that CT has a sensitivity of about 79% and specificity of 87% for identifying pelvic masses as benign or malignant [5]. Ultrasound has a sensitivity of around 66% to 78% when determining the tissue of origin [6].

In comparison, MRI offers much better soft tissue contrast and the ability to view structures in multiple planes [7]. It has been shown to have up to 97% overall accuracy in characterizing pelvic masses and correctly identifies the tissue of origin in about 94% of cases. In evaluating adnexal masses, contrast-enhanced MRI has demonstrated near 100% sensitivity and about 94% specificity [8]. While ultrasound may have similar sensitivity, its specificity is often much lower. 

In this patient’s case, where imaging showed a solid and vascular pelvic mass with an unclear origin, MRI might have helped distinguish whether the mass came from the reproductive organs or the gastrointestinal tract [9]. The imaging appearance of an extrauterine leiomyoma can closely resemble that of ovarian fibromas or gastrointestinal stromal tumors, as all of these entities often appear as solid, hypoechoic, and well-circumscribed masses, making them difficult to distinguish on imaging alone [10]. MRI could have provided clearer separation of nearby structures and better defined the source of the mass, potentially allowing for more accurate preoperative planning.

The differential diagnosis included ovarian fibroma, ovarian neoplasm, gastrointestinal stromal tumor (GIST), and mesenteric leiomyoma. A definitive diagnosis was only possible after surgical removal and histopathologic analysis. The initial findings showed a spindle cell tumor, which required further evaluation and immunohistochemical staining to confirm it as a benign leiomyoma. This case illustrates the importance of keeping a broad differential when evaluating pelvic masses, especially when imaging findings are inconclusive.

## Conclusions

This case illustrates the diagnostic challenges of identifying extrauterine leiomyomas that mimic adnexal masses on imaging. Although CT and ultrasound provided structural details, they could not determine the true origin of the mass. MRI may have offered improved soft tissue contrast and anatomical clarity, potentially aiding in preoperative planning. Ultimately, surgical exploration and histopathological analysis were necessary for diagnosis. This case emphasizes the importance of maintaining a broad differential when evaluating pelvic masses and considering non-gynecologic sources, such as gastrointestinal or mesenteric tumors. A multidisciplinary approach incorporating advanced imaging, surgery, and pathology is essential for accurate diagnosis and optimal patient care.

## References

[1] Kumar V, Abbas AK, Aster JC (2018). Robbins Basic Pathology.

[2] Chin H, Ong XH, Yam PK, Chern BS (2014). Extrauterine fibroids: a diagnostic challenge and a long-term battle. BMJ Case Rep.

[3] Nikolic O, Basta Nikolic M, Spasic A, Otero-Garcia MM, Stojanovic S (2019). Systematic radiological approach to utero-ovarian pathologies. Br J Radiol.

[4] Zuhdy M, Belal K, Shata M (2023). A large extraluminal ascending colon leiomyoma mimicking a uterine fibroid: a case report and literature review. Surg Gastroenterol Oncol.

[5] Jung SE, Lee JM, Rha SE, Byun JY, Jung JI, Hahn ST (2002). CT and MR imaging of ovarian tumors with emphasis on differential diagnosis. Radiographics.

[6] Kinkel K, Hricak H, Lu Y, Tsuda K, Filly RA (2000). US characterization of ovarian masses: a meta-analysis. Radiology.

[7] Saini A, Dina R, McIndoe GA, Soutter WP, Gishen P, deSouza NM (2005). Characterization of adnexal masses with MRI. AJR Am J Roentgenol.

[8] Thomassin-Naggara I, Aubert E, Rockall A, Jalaguier-Coudray A, Rouzier R, Daraï E, Bazot M (2013). Adnexal masses: development and preliminary validation of an MR imaging scoring system. Radiology.

[9] Spencer JA, Ghattamaneni S (2010). MR imaging of the sonographically indeterminate adnexal mass. Radiology.

[10] Radswiki T, Moore C, Sattar A (2025). Ovarian fibroma. Reference article. Radiopaedia.org.

